# A novel Trmt5-deficient zebrafish model with spontaneous inflammatory bowel disease-like phenotype

**DOI:** 10.1038/s41392-023-01318-6

**Published:** 2023-02-27

**Authors:** Qiong Zhao, Hui Chang, Jing Zheng, Ping Li, Lidan Ye, Ruolang Pan, Di Li, Jian-Zhong Shao, Robert Chunhua Zhao, Ye Chen

**Affiliations:** 1 Department of Genetics, and Department of Genetic and Metabolic Disease, The Children’s Hospital, Zhejiang University School of Medicine, National Clinical Research Center for Child Health, Hangzhou, 310052 China; 2 Institute of Basic Medical Sciences, Chinese Academy of Medical Sciences, School of Basic Medicine, Peking Union Medical College, Beijing, 100005 China; 3 College of Life Sciences, Key Laboratory for Cell and Gene Engineering of Zhejiang Province, Zhejiang University, Hangzhou, 310052 China; 4 Zhejiang Provincial Key Laboratory of Genetic and Developmental Disorders, Institute of Genetics, Zhejiang University, Hangzhou, 310052 China; 5 Key Laboratory for Food Microbial Technology of Zhejiang Province, Zhejiang Gongshang University, Hangzhou, China; 6 Institute of Bioengineering, College of Chemical and Biological Engineering, Zhejiang University, Hangzhou, 310027 China; 7 Zhejiang Provincial Key Laboratory of Cell‐Based Drug and Applied Technology Development, Institute for Cell‐Based Drug Development of Zhejiang Province, Hangzhou, China

**Keywords:** Experimental models of disease, Gastrointestinal diseases

**Dear Editor**,

Inflammatory bowel disease (IBD), a complex syndrome characterized by chronic inflammation of the gastrointestinal tract, is considered a global health problem, especially prevalent in western developed countries and with accelerating incidence in the developing world over the last decade.^1^ To date, the primary etiology of IBD remains elusive. Accumulated evidence suggests a significant connection between intestinal inflammation and mitochondrial dysfunction.^2,3^ Abnormalities in the structure and function of mitochondria have been observed in IBD patients and experimental models.^4^ However, the pathophysiological roles of various mitochondrial components in IBD are mainly unknown, necessitating the development of novel animal models to delineate pathogenic genes and unravel related mechanisms.

Herein, we generated a novel zebrafish IBD model based on the tRNA methyltransferase 5 (*TRMT5*) gene that was reduced expressed in mucosal biopsies from IBD patients (Supplementary Fig. 1). Multiple alignments of zebrafish Trmt5 with its homologs of other organisms revealed broad protein sequence conservation (Supplementary Fig. 2). We generated *trmt5* knockout zebrafish lines using CRISPR-Cas9 approach (Supplementary Fig. 3). Unlike embryonic lethal *Trmt5*^−/−^ mice, *trmt5*^−/−^ zebrafish survived. The gut of *trmt5*^−/−^ was comparable to wild-type siblings by 5 dpf (Fig. 1a) when the intestine was fully developed and functional, and exotrophic nutrition began in zebrafish. It is probably due to the high abundance of maternally expressed *trmt5* in early larval stages (Supplementary Fig. 4). Afterward, the *trmt5*^−/−^ mutants gradually exhibited defects, including shorter body length, reduced dorsal-ventral size, and significantly enhanced mortality (Supplementary Fig. 5).

**Fig. 1:**
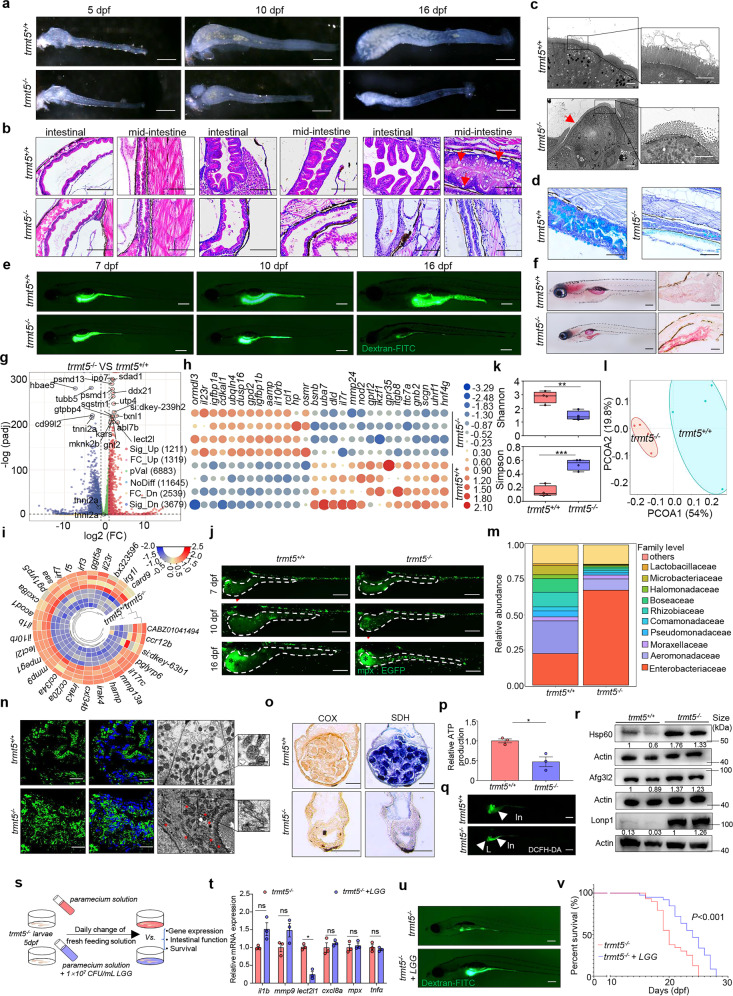
A novel Trmt5-deficient zebrafish model with spontaneous inflammatory bowel disease-like phenotype. **a** Representative anatomical diagrams of zebrafish guts at 5 dpf, 10 dpf, and 16 dpf. Scar bar = 200 μm. **b** Representative hematoxylin-eosin staining of sagittal sections corresponding to the intestinal bulb or mid-intestine of two genotypes larvae at different time points. The red arrows indicate goblet cells in the mid-intestine. Scar bar = 100 μm. **c** TEM analysis of the intestinal epithelium of *trmt5*^−/−^ and *trmt5*^*+/+*^ at 16 dpf. *trmt5*^−/−^ IHCs exhibited shorter microvilli (red arrow) than the wild-type controls. Scar bar = 4 μm. **d** Alcian blue staining of sagittal sections of middle intestine at 16 dpf. Scar bar = 100 μm. **e** In vivo imaging of zebrafish digestive organ after incubation with 1% dextran-FITC for 4 h at 28.5 °C. Scar bar = 200 μm. **f** Oil Red O (ORO) staining for neutral lipid accumulation at 16 dpf. The left panel is a whole-mount of ORO staining, and the right panel is cryo-sections. Scar bar = 200 μm. **g** Volcano plot of differentially expressed genes (DEGs). Red and blue plots indicate upregulated and downregulated DEGs, respectively. **h** Heatmap of IBD-related DEGs. **i** Circular heatmap of 29 upregulated immune-related genes in *trmt5*^−/−^ compared to *trmt5*^*+/+*^. **j** Whole-mount of *trmt5*^−/−^ and *trmt5*^*+/+*^ *Tg (mpx: EGFP)* transgenic larvae at 7 dpf, 10 dpf, and 16 dpf. *Tg (mpx: EGFP)*, is a neutrophil-specific transgenic line. The clearly scattered green dots (indicated by red arrow) represent the neutrophils, while the diffuse green signal (showed by black asterisk) is background noise from the zebrafish abdominal cavity. Scar bar = 200 μm. **k** Alpha diversity comparison of the microbiota in *trmt5*^−/−^ and *trmt5*^*+/+*^ intestine. *p* indicates the significance (**p < 0.01, ***p < 0.001). **l** UniFrac principal coordinate analyses (PCoA) analysis. **m** Metagenome analysis of the intestinal microbiota of *trmt5*^−/−^ and *trmt5*^*+/+*^ at the family levels. **n** Comparison of the mitochondrial morphology between *trmt5*^−/−^ and *trmt5*^*+/+*^ intestine at 16 dpf by *Tg (Xla.Eef1a: MLS-EGFP)* transgenic larvae and TEM. *Tg (Xla.Eef1a: MLS-EGFP)* expresses mitochondrially targeted EGFP. Nuclei were co-stained with DAPI (blue). Scar bar = 20 μm in confocal images, and scar bar = 0.5 μm in TEM images. **o** COX/SDH staining of intestine at 16 dpf. Scar bar = 100 μm. **p** The relative total ATP production at 16 dpf. **q** ROS production was examined by DCFH-DA staining at 16 dpf. In: intestine; L: liver. Scar bar = 200 μm. **r** The expression levels of proteins involved in UPR^mt^, including Hsp60, Afg3l2, and Lonp1. **s** Schematic representation of the experimental design. **t** mRNA expression of pro-inflammatory genes in two groups of zebrafish at 16 dpf. **u** The swallow activity was detected by FITC-labeled dextran at 16 dpf. **v** The survival was significantly extended after *LGG* daily feeding in *trmt5*^−/−^ zebrafish

We performed anatomical and histological examinations at various time points and observed that *trmt5*^−/−^ zebrafish gradually developed intestinal defects during late larval stages. Reduced intestinal epithelium was observed in *trmt5*^−/−^ guts with disappearance of intestinal-fold architecture, flattened epithelium, disorganized localization of cell nuclei, and reduced goblet cells (Fig. 1b). We observed disrupted and shortened microvilli, defective tight and adherent junctions of intestinal epithelial cells (IECs) in *trmt5*^−/−^ intestinal epithelium (Fig. 1c, Supplementary Fig. 6a–d), and decreased goblet cell numbers in *trmt5*^−/−^ middle intestine (Fig. 1d). Next, we performed swallowing activity assay and Oil Red staining, respectively. The results showed a significantly reduced dextran-FITC in *trmt5*^−/−^ mutant at 10 dpf (~60% reduction) and 16 dpf (~90% reduction) (Fig. 1e). An apparent ORO staining signal for lipid was found in the foregut region of *trmt5*^−/−^ mutants at 16 dpf, indicating disrupted intestinal lipid metabolism (Fig. 1f). Meanwhile, a significantly increased level of apoptosis was observed in the *trmt5*^−/−^ intestine (Supplementary Fig. 6e–g). Together, these data suggested that the mutant phenotype is not primarily an early developmental defect but reflects a requirement for Trmt5 in maintaining intestinal function at later larval stages.

To further identify the hallmark features of human IBD in the established *trmt5*^−/−^ zebrafish, we applied RNA-sequencing analyses between *trmt5*^−/−^ mutants and wild-type siblings at 16 dpf. 1211 up-regulated and 3679 down-regulated differently expressed genes (DEGs) were identified in *trmt5*^−/−^ mutants (Fig. 1g). Dysregulation of intestinal-related and IBD-related genes, including intestinal barrier-related genes, bacterial sensing and autophagy-related genes, and inflammatory response genes, etc., were observed *trmt5*^−/−^ mutants (Fig. 1h, Supplementary Fig. 7a–d). Moreover, GO/KEGG analyses indicated that the down-regulated DEGs are primarily involved in oxidation-reduction process, transmembrane transport, proteolysis, ion transport, and lipid metabolic process, which are activated in normal adult zebrafish intestine (Supplementary Fig. 7e); and the up-regulated DEGs were significantly enriched in the process of immune response and bacterial and viral infections (Fig. 1i, Supplementary Fig. 7f, g), indicating the activation of the immune response. Indeed, we showed that the inflammatory response was gradually activated along with the increasing severity of the intestinal defects (Supplementary Fig. 7h, i). Furthermore, we proved the deletion of *trmt5* leads to recruitment of neutrophils in intestine at later larval stages using a neutrophil-specific transgenic *Tg* zebrafish line, as well as macrophages and Natural Killer (NK) cells by qRT-PCR analysis (Fig. 1j, Supplementary Fig. 8).

Intestinal microbiome disorders were found to be associated with IBD pathogenesis.^5^ We further investigated whether the intestinal microorganisms are involved in the *trmt5* deletion-induced pathogenesis by 16 s rDNA amplicon sequencing. Results showed that the intestinal microbial diversity in *trmt5*^−/−^ zebrafish was significantly reduced (Fig. 1k). The PCoA-plot based on weighted Unifrac indicated a significant separation between the microbiota of the two groups (Fig. 1l). The intestinal microbiome composition in mutant zebrafish was changed at both the class (Supplementary Fig. 9a) and family levels (Fig. 1m). STAMP analysis revealed that *trmt5*^−/−^ mutants exhibited an increased relative abundance in Gamma-proteobacteria, one of the main classes of Gram-negative pathogenic bacteria expanded under inflamed conditions, and reduced in Alpha-proteobacteria. Significantly, Enterobacteriaceae, overgrowing under host-mediated inflammation conditions, was highly enriched in *trmt5* mutants at the family level. Besides, the relative abundance of *Lactobacillaceae*, known as probiotic strains and inhibiting inflammation, was reduced in *trmt5*^−/−^ mutants (Supplementary Fig. 9b, c). Together, these findings indicated that the *trmt5*^−/−^ zebrafish exhibited some hallmark features of IBD, which more likely represents human Crohn’s disease.

Then we determined mitochondrial signaling and concomitant changes in *trmt5*^*−/−*^ mutants to explore the potential mechanism. RNA-seq revealed that 135 mitochondrial-related genes were expressed differently in *trmt5*^*−/−*^ mutants, among which 91 were downregulated and 44 were upregulated (Supplementary Fig. 10a). GO analysis indicated that the most significant number of genes enriched in oxidation-reduction process (Supplementary Fig. 10b). The protein expression levels of representative OXPHOS subunits, including Nd6 (subunits of respiratory Complex I; CI), Uqcrc2 (CIII), Co2 (CIV), as well as Atp8 (CV), were all declined in *trmt5*^−/−^ mutants (Supplementary Fig. 10c). Abnormal and elongated morphology of mitochondria was observed in intestinal epithelial cells of *trmt5*^−/−^ mutants (Fig. 1n, Supplementary Fig. 10d, e). Moreover, the enzymatic activities of COX and SDH were decreased (Fig. 1o), suggesting OXPHOS deficiency in *trmt5*^−/−^ intestine. Consequently, reduced production of ATP and overproduction of ROS were recorded in *trmt5*^−/−^ intestine (Fig. 1p, q). In addition, mitochondrial unfolded protein response (UPR^mt^) simulation, an integral aspect of IBD pathologies, was found in *trmt5*^−/−^ mutants (Fig. 1r). In particular, mitochondrial heat shock protein (Hsp60), which indicates UPR^mt^ and is considered as a putatively significant driver of IBD, was significantly up-regulated in *trmt5*^−/−^ zebrafish. These data indicated that deletion of *trmt5* caused mitochondrial dysfunction in the zebrafish intestine, which may be responsible for the spontaneously developed IBD-like phenotype.

Probiotics have demonstrated protective effects in case of intestinal inflammation. Indeed, we showed the protective effect of *Lactobacillus GG* (*LGG*) in *trmt5*^−/−^ larvae, which partially alleviated the IBD-like phenotype (Fig. 1s), including significantly reduced expression of pro-inflammatory gene *lect2l* (Fig. 1t), dramatically improved swallow activities (Fig. 1u), and extended median survival (*p* < 0.01, Fig. 1v). Besides, the IBD-like phenotype of *trmt5*^−/−^ larvae was significantly alleviated by MitoTempo (mitochondrial antioxidant) but not dexamethasone (glucocorticoid with anti-inflammatory properties) (Supplementary Fig. 11). In summary, the present study generated the first Trmt5 deficiency vertebrate animal line in zebrafish with spontaneous-develop pathological features of human IBD, including epithelial disruption, goblet cells depletion, and immune system overactivation. In addition, akin to IBD treatment strategies, we showed that the mutant phenotype was partially alleviated after probiotic or MitoTempo administration. Thus, we propose that the *trmt5*^−/−^ zebrafish may serve as a disease model for studying IBD pathogenesis and a platform for developing and evaluating potential therapeutic interventions. Our findings suggest that focusing on mitochondrial dysfunction may have broad translational utilities in patients with IBD.

## Supplementary information

Supplementary material [SIGTRANS-07770R]-clear

## Data Availability

All data and materials are presented in the main manuscript or supplementary materials and are available on request. RNA and 16 s rDNA sequencing data were deposited into Sequence Read Archive (SRA) with the Bio-project ID of PRJNA833575 and PRJNA855381.
