# Supplementary material for: A novel Trmt5-deficient zebrafish model with spontaneous inflammatory bowel disease-like phenotype
Source: Signal Transduct Target Ther. 2023 Feb 27;8:86. doi: 10.1038/s41392-023-01318-6 (PMC9971238; doi:10.1038/s41392-023-01318-6)
Supplement: Supplementary file 1 — Supplementary material [SIGTRANS-07770R]-clear [file 41392_2023_1318_MOESM1_ESM.docx]

Supplementary Materials for

**A novel Trmt5-deficient zebrafish model with spontaneous inflammatory bowel disease-like phenotype**

Qiong Zhao^1,4#^, Hui Chang^1,4#^, Jing Zheng^1,4^, Ping Li^5^, Lidan Ye^6^, Ruolang Pan^7^, Di Li^2^,

Jian-Zhong Shao^3^*, Robert Chunhua Zhao^2^*, Ye Chen^1,4^*

Correspondence to: Ye Chen (yechency@zju.edu.cn), Robert Chunhua Zhao (zhaochunhua@ibms.pumc.edu.cn), Jian-Zhong Shao (shaojz@zju.edu.cn)

**This PDF file includes:**

Materials and Methods

Figures. S1 to S11

Table. S1

## Materials and Methods

**Zebrafish strains and maintenance**

Tuebingen (Tu) wild-type strain, transgenic *Tg (Xla.Eef1a: MLS-EGFP)* expressing mitochondrially targeted enhanced green fluorescent protein (EGFP), and neutrophil-specific transgenic *Tg (mpx: EGFP)* zebrafish (*Danio rerio*) were used for this investigation. The animal protocols used in this investigation were approved by the Zhejiang University Institutional Animal Care and Use Committee (NO: 18020). All fish were kept in recirculating water at 28.5°C and fed with commercial pellets daily to 0.7% of their body weight. Embryos were reared at 28.5°C according to standard protocols^1^. Embryos were staged by hours post-fertilization (hpf) and days post fertilization (dpf)^2^.

**Generation and identification of *trmt5* knockout zebrafish**

*trmt5* knockout zebrafish were generated by CRISPR-Cas9 technology. The CRISPR-Cas9 design tool ([http://zifit.partners.org](http://zifit.partners.org/)) was adopted to select specific targets and minimize off-target effects. sgRNAs were transcribed from the chemically synthesized and PCR-amplified templates using a T7 high-efficiency transcription kit (Transgene, China) according to the manufacturer’s instructions. Then, Cas9 protein (1 mM, EnGen, New England Biolabs, UK) and gRNA (200 ng/μL) were co-injected into one-cell-stage wild-type embryos. Those injected embryos were plated in E3 medium (5 mM NaCl, 0.17 mM KCl, 0.33 mM CaCl_2_, 0.33 mM MgSO_4_, 0.0003% methylene blue, pH 7.4 with sodium bicarbonate) and incubated at 28.5 °C. For genotyping, genomic DNAs of injected embryos were extracted at 50 hpf for *trmt5* gene amplification using the following two primers: F: TCAGTTTGTTTTTGCTTGATTCGAT; R: GTCGAGGGTTCCAGTACACA. The fragments were cloned using the TA Cloning Kit (TAKARA, Japan) and then sequenced.

**Whole-mount in situ hybridization (WISH)**

Probes were synthesized with digoxigenin (DIG)-labeled antisense RNA probes specific to zebrafish *trmt5* and *fabp2* using the primers depicted in [Supplemental Table S1](https://www.ncbi.nlm.nih.gov/pmc/articles/PMC8096277/#sup1). WISH was carried out as previously described^3,4^. Zebrafish embryos from various ages of post-fertilization were dechlorinated in 2 mg/ml pronase (Roche, Switzerland) in E3 medium and fixed at 4°C in fresh 4% paraformaldehyde (PFA, BBI Life Science, China) in phosphate-buffered saline (PBS, BBI Life Science, China) overnight. Briefly, embryos were permeabilized in 40 μg/ml proteinase K (BBI Life Science, China) for 20 min, and probes were used at a 1 μg/ml concentration in the hybridization solution. After the hybridization procedure, embryos were washed extensively in PBS with 0.1% Tween 20 (BBI Life Science, China), re-fixed in 4% paraformaldehyde, and transferred to 70% glycerol (BBI Life Science, China). Stained embryos were visualized using a stereoscopic microscope (SMZ18, Nikon).

**Zebrafish swallowing activity assay**

Dextran-FITC (MW 4000, 46944, Sigma) was adopted to determine the swallowing activity of the zebrafish intestine^5^. Larvae were immersed in E3 medium with 1% dextran-FITC for 4 h at 28.5°C, then imaged under an epifluorescence microscope (SMZ25; Nikon, Tokyo, Japan).

**Histological studies**

For hematoxylin and eosin (H&E) staining, zebrafish were anesthetized and fixed in Bouin’s Fluid (Solabio, Beijing, China) at 4°C overnight. Samples were then dehydrated, infiltrated, embedded in paraffin, and sliced into 4 μm thick by pathologic microtome (RM2016, Leica). Sections were stained with hematoxylin and eosin (H&E, Beyotime, China) solution. For alcian blue staining, the paraffin-embedded slides were stained in alcian blue solution (Solabio, Beijing, China) for 30 mins. For immunofluorescent staining, zebrafish were fixed in 4% paraformaldehyde at room time for 20 mins, then incubated in 30% sucrose solution at 4°C overnight. The samples were embedded in the OCT compound at -80°C, and 10 μm cryo-sections were made using a CM1950 cryostat (Leica, Germany). The slides were stained with phalloidin-iFluor 555 (Abcam, UK) and TUNEL-FITC (Beyotime, China), and the nucleus was visualized using 4′6-diamidino-2-phenylindole (DAPI, Beyotime, China). Enzyme histochemistry (EHC) analysis for succinate dehydrogenase (SDH) and cytochrome c oxidase (COX) in the frozen-sections were performed as previously described^6^. Briefly, zebrafish were fixed in 4% PFA at room temperature for 5 min and then embedded in the OCT compound at -80°C. 10 μm cryo-sections were stained with COX (5 mM phosphate buffer, pH 7.4, containing 0.1% 3,3′-diaminobenzidine (DAB), 0.1% Cytochrome c, 0.02% catalase) and SDH (5 mM phosphate buffer, pH 7.6, containing 5 mM EDTA, 1 mM potassium cyanide (KCN), 0.2 mM phenazine methosulfate (PMS), 50 mM succinic acid, 1.5 mM nitro blue tetrazolium (NBT)) solution following the standard protocols. Images were obtained using a confocal microscope or light microscope (OLYMPUS, FV1000).

**Transmission electron microscopy (TEM) analysis**

Larvae at 16 dpf were fixed in 2.5% glutaraldehyde (Sigma, USA), embedded in Epon 812 (Shell Chemical, USA), and cut into 70-90 nm thick slices using UC7 ultramicrotome (Leica, Heerbrugg, Switzerland), then stained with uranyl acetate and lead citrate. The images of myocardial ultra-structure were captured using a Hitachi-7650 transmission electron microscope (Hitachi, Tokyo, Japan).

**RNA isolation and quantitative real-time PCR**

Total RNAs were extracted using TRIzol reagent (Takara) following the manufacturer’s protocol and transcribed into cDNA using the PrimeScript™ RT Reagent Kit (Takara, Japan). Quantitative real-time PCR analyses were performed using an Hieff^®^ qPCR SYBR Green Master Mix (Yeasen, China) on the Applied Biosystems Prism 7500 System with gene-specific primers. *elfα* was used as the endogenous control. The 2^-ΔΔCT^ method was selected for transcript quantification. Detailed primer sequences are provided in [Supplemental Table S1](https://www.ncbi.nlm.nih.gov/pmc/articles/PMC8096277/#sup1).

**RNA sequencing analysis**

Total RNAs were extracted from a pool of 10 larvae at 16 dpf using the TRIzol-based standard procedure (Takara). cDNA libraries were generated using NEB Next Ultra Directional RNA Library Prep Kit (NEB) and sequenced on an Illumina Hiseq2500 at LC Bio (Zhejiang, China). Genes with an adjusted *p*-value < 0.05 and |log_2_FC| > 1 were assigned as differentially expressed using the DESeq2 R package (1.20.0). Gene Ontology (GO) enrichment analysis of differentially expressed genes was implemented by the cluster Profiler R package, in which gene length bias was corrected. GO terms with adjusted *p*-value < 0.05 were considered significantly enriched by differential expressed genes. Kyoto encyclopedia of genes and genomes (KEGG) pathway analysis was performed using the Cluster Profiler R package. The RNA sequencing (RNA-Seq) data were deposited into Sequence Read Archive (SRA) with the Bio-project ID of PRJNA833575.

**16s rDNA sequencing analysis**

Total DNAs were isolated from a pool of 10 larvae’s guts at 16 dpf using a DNA extraction kit (Vazyme, China) following the manufacturer’s protocol. DNAs were amplified by targeting the V3-V4 region of the 16s rDNA to construct the sequencing library. PE250/FE300 double-terminal sequencing was performed according to Illumina MiSeq/Novaseq instrument instructions to obtain original sequencing Data (Pass Filter Data) (KAITAI-BIO, China). The reference database is Silva138, and the sequence clustering analyses were conducted using VSEARCH (1.9.6) (sequence similarity is set to 97%). The relative abundance of intestinal operational taxonomic units (OTUs) was analyzed using the RDP Classifier (Ribosomal Database Program) Bayesian algorithm. The community composition of each sample was calculated at different species classification levels. The sequencing data were deposited into Sequence Read Archive (SRA) with the Bio-project ID of PRJNA855381.

**Western blotting**

Fish were sacrificed after anesthesia and lysed in RIPA reagent (Beytime, China). Boiled lysates (in Laemmli buffer) were separated on 12% polyacrylamide gels, and proteins were then transferred to a polyvinyl difluoride (PVDF, Millipore, Massachusetts, USA) membrane for immunoblotting with primary antibodies. Following subsequent incubation with secondary antibodies, signals were visualized using an ECL system (CWBIO, China) and quantified by Image J software. Primary antibodies used include Hsp60 (ab46798), Nd6 (ab81212), Uqcrc2 (ab203832), Trmt5 (18255-1-AP), Lonp1 (15440-1-AP), Afg3l2 (14631-1-AP), Gapdh (60004-1-Ig), Beta Actin (66009-1-Ig), Co2 (55070-1-AP), and Atp8 (26723-1-AP).

**Probiotics treatment**

Probiotics have demonstrated protective effects in case of intestinal inflammation^7^. Here, we tested the impact of *Lactobacillus GG* (*LGG*) as a representative in *trmt5^-/-^* larvae^8^. *LGG* is a well-known probiotic strain which showed significant protective effects against murine colitis. *Lactobacillus GG* (*LGG*) was obtained from Danisco Co., Ltd. (Kunshan, China). Genotyped *trmt5^-/-^* larvae at 5 dpf were randomly divided into two groups, namely *trmt5^-/-^* and *trmt5^-/^*+ LGG group *^-^*. The lyophilized LGG powder was suspended in the larval media at 10^7^ CFU/mL. The two groups of fish were fed twice a day with the same volume of paramecium solution with or without LGG. The solution was exchanged daily to maintain *LGG* concentrations, and the general condition and mortality of trmt5^-/-^ larvae were monitored daily. The intestinal swallow activity was determined.

**Statistical analysis**

Statistical analysis was carried out based on at least three independent experiments using unpaired two-tailed Student’s t-test in the GraphPad Prism 8 program. The error bars indicate two standard deviations of the means (SEM). Differences were considered significant at *p*-value of < 0.05.

**References：**

1 Westerfield, M. *The Zebrafish Book. A Guide for The Laboratory Use of Zebrafish (Danio rerio)*. 4th edn, (University of Oregon Press, Eugene, 2000).

2 Kimmel, C. B. *et al.* Stages of embryonic development of the zebrafish. *Dev Dyn*. **203**, 253-310, (1995).

3 Moens, C. Whole mount RNA in situ hybridization on zebrafish embryos: mounting. *CSH Protoc*. **3**, 5038, (2008).

4 Moens, C. Whole mount RNA in situ hybridization on zebrafish embryos: hybridization. *CSH Protoc*. **3**, 5037, (2008).

5 Zhao, S. *et al.* Deficiency in class III PI3-kinase confers postnatal lethality with IBD-like features in zebrafish. *Nat Commun*. **9**, 2639, (2018).

6 Jin, X. *et al.* An animal model for mitochondrial tyrosyl-tRNA synthetase deficiency reveals links between oxidative phosphorylation and retinal function. *J Biol Chem*. **296**, 100437, (2021).

7 Macpherson, A. J. & Harris, N. L. Interactions between commensal intestinal bacteria and the immune system. *Nat Rev Immunol*. **4**, 478-485, (2004).

8 Li, Y. *et al.* Inhibitory Effects of the Lactobacillus rhamnosus GG Effector Protein HM0539 on Inflammatory Response Through the TLR4/MyD88/NF-кB Axis. *Front Immunol*. **11**, 551449, (2020).


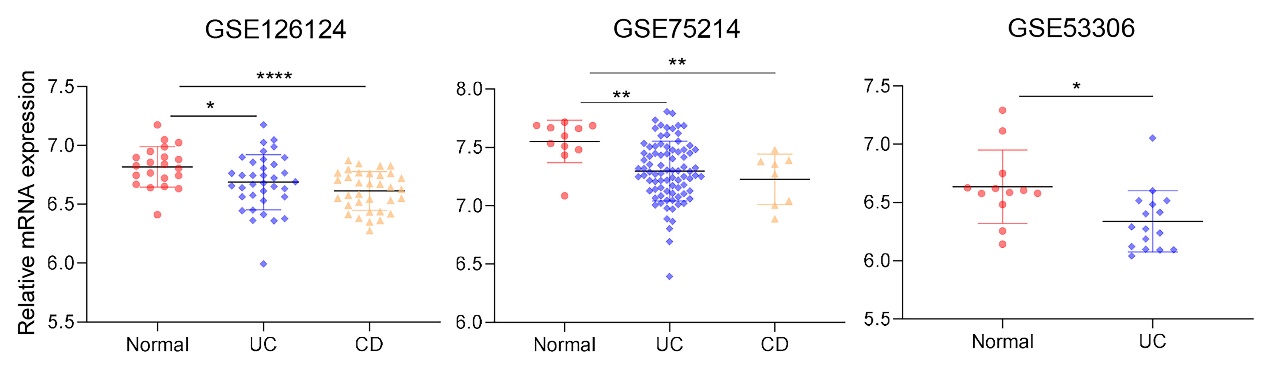


**Supplementary Figure 1.** Comparison of TRMT5 mRNA expression levels in colonic tissues between IBD patients and healthy controls in GSE126124, GSE75214, and GSE53306.


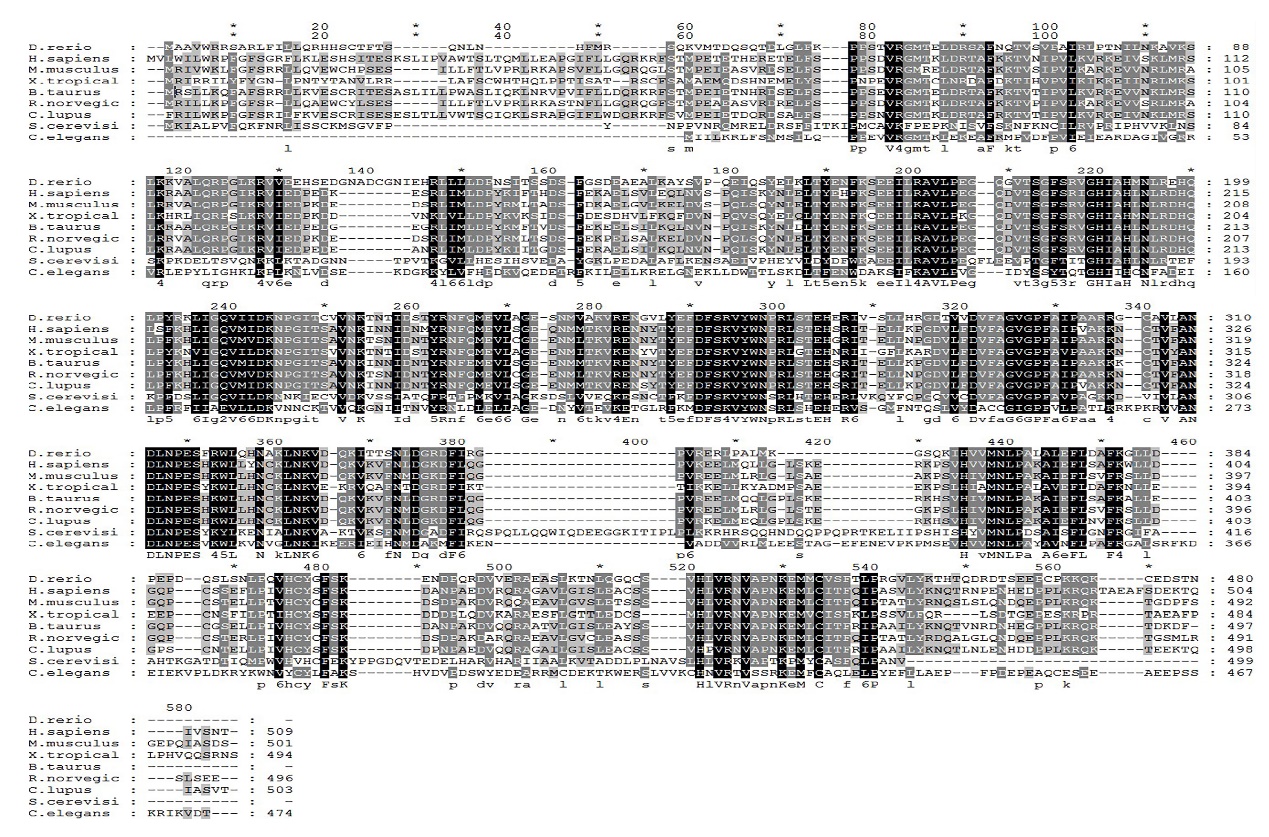


**Supplementary Figure 2.** Amino acid sequence multiple alignments of zebrafish Trmt5 with its homologs of other organisms, including *Homo sapiens*, *Mus musculus*, *Xenopus tropicalis*, *Bos Taurus*, *Rattus norvegicus*, *Canis lupus*, *Saccharomyces cerevisiae,* and *Caenorhabditis elegans* using ClustalW.


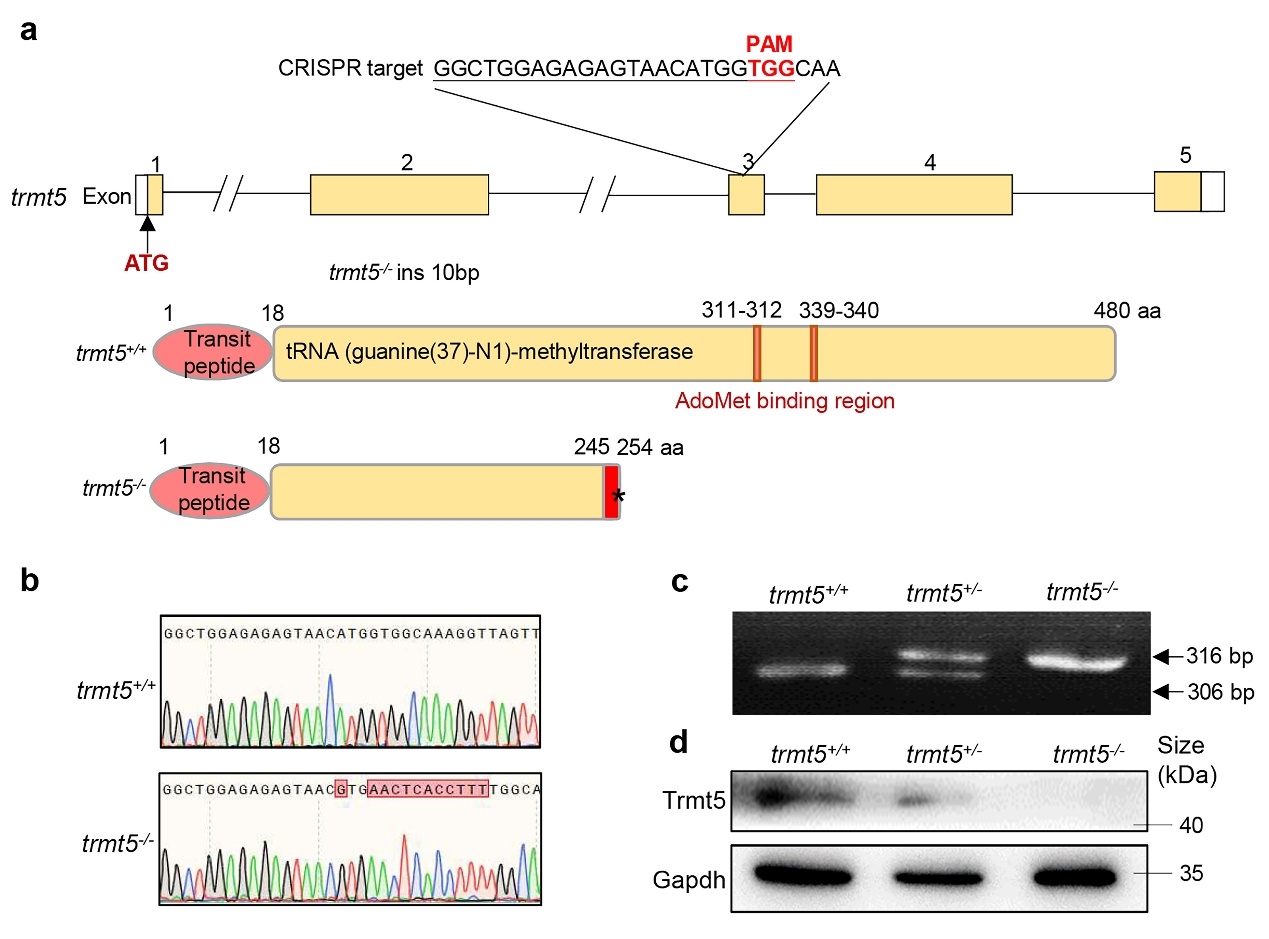


**Supplementary Figure 3. Generation of *trmt5* knockout zebrafish model. a** Schematic drawing of CRISPR/Cas9 gene editing for *trmt5* locus and comparing wild-type and truncated proteins generated from the mutations. The gRNA target site is in exon 3, and the PAM region is marked red. A 10 bp insertion mutation was induced and caused a premature stop at codon 254 in *trmt5^-/-^* mutants. The black asterisk indicated a premature stop codon. **b, c** Genotyping of *trmt5^-/-^* mutants by Sanger sequence and DNA polyacrylamide gel electrophoresis. **d** Western blot analysis revealed a successful knockout of Trmt5 in zebrafish. GAPDH is adopted as the loading control.


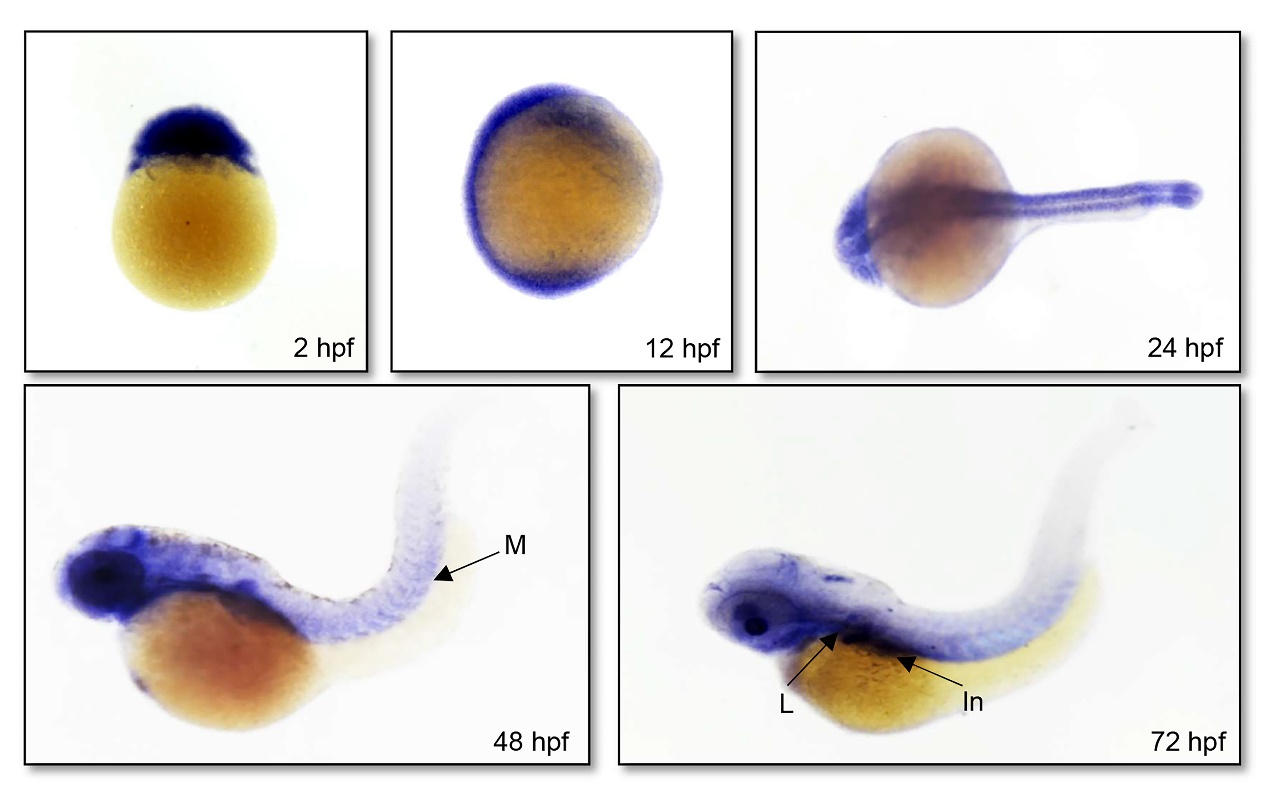


**Supplementary Figure 4.** The spatiotemporal expression pattern of *trmt5* in zebrafish embryos. Whole-mount in situ hybridization (WISH) analysis of *trmt5* in wild-type larvae at different time points. M: muscle, L: liver, In: intestine.


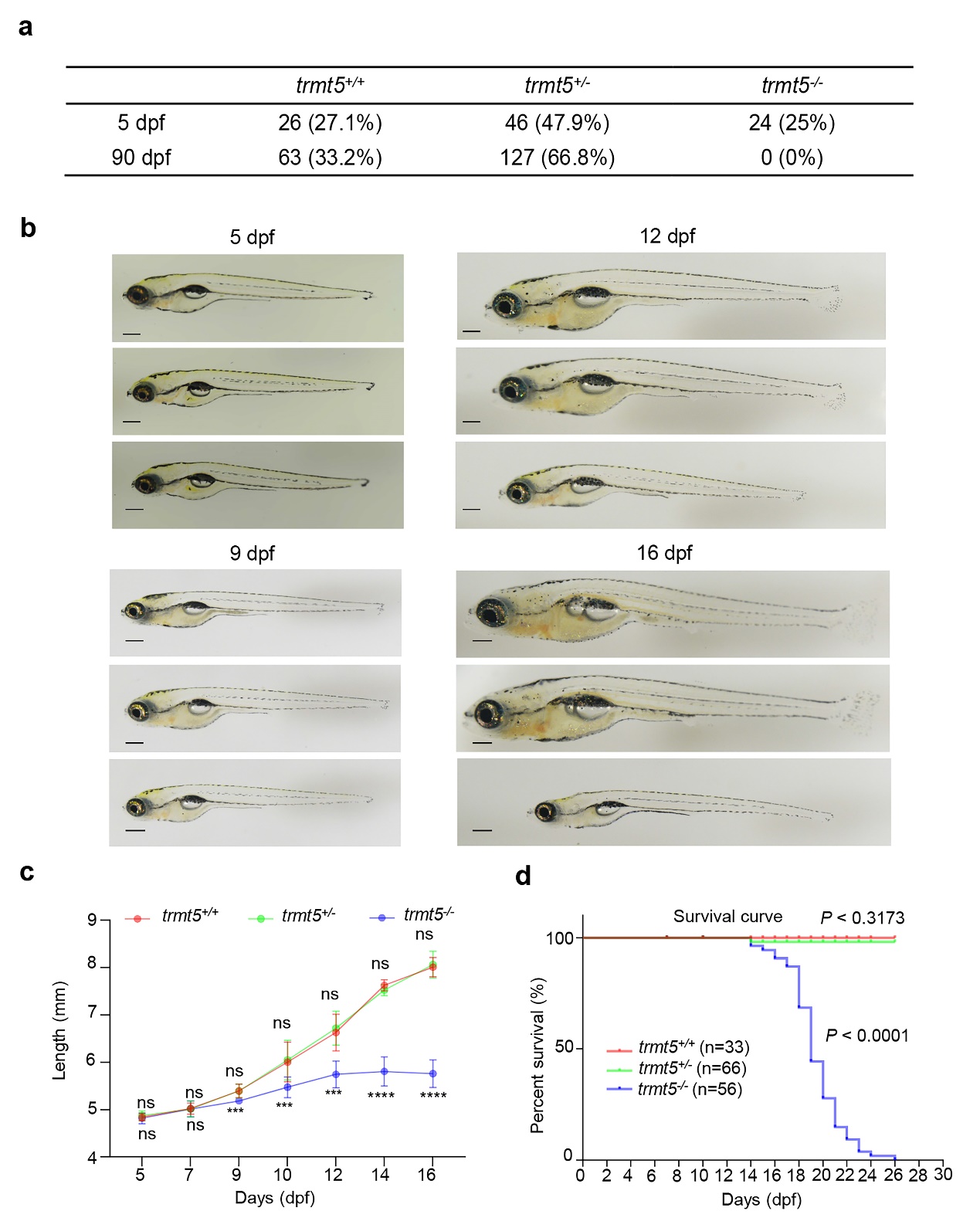


**Supplementary Figure 5. *trmt5^-/-^* larvae were lethal**. **a** Genotype ratio of zebrafish at 5 dpf and 90 dpf. **b** Representative images of the morphology of *trmt5^-/-^* and *trmt5^+/+^* larvae under a light microscope at 5 dpf, 9 dpf, 12 dpf, and 16 dpf. Scar bar = 200 μm. **c** Quantification of body length of larvae at different time points. n > 10 per group, *p* indicates the significance (****p* < 0.001, ****p* < 0.0001, ns, no significant difference). **d** Survival curves of three genotypes of larvae.


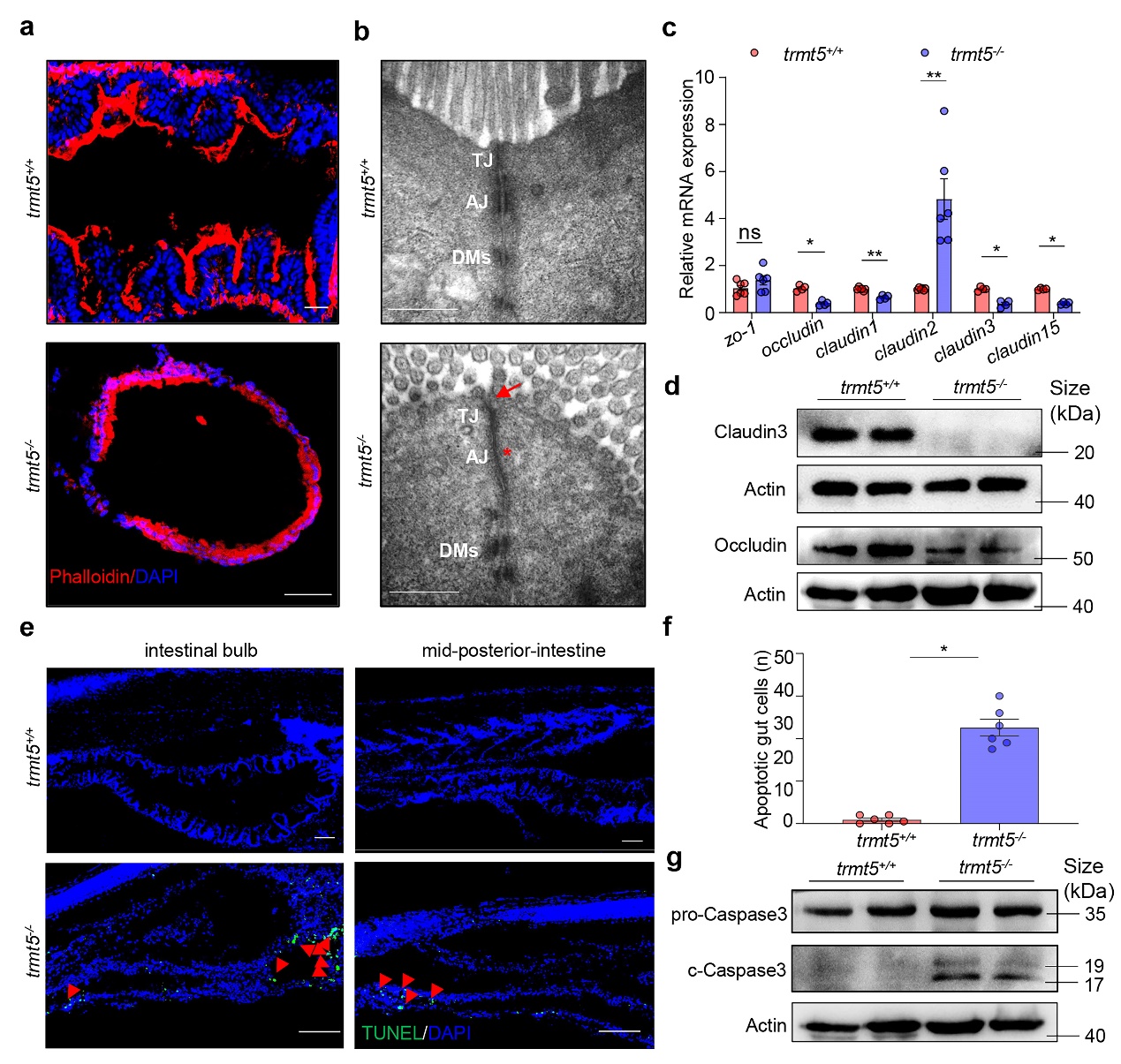


**Supplementary Figure 6. a** Confocal images of intestinal cross-sections stained by phalloidin-iFluor 555 at 16 dpf. Scar bar = 100 μm. **b** TEM analyses of cell junction between IECs. TJ, tight junction, AJ, adherent junction, DMs, desmosomes. The red arrow indicated a defective tight junction, and the red asterisk showed a disrupted adherent junction. **c** Relative mRNA expression of tight junction-related genes was analyzed at 16 dpf. **d** The expression of tight junction proteins. Actin is employed as a loading control. **e** Representative images of TUNEL staining of sagittal sections. Red arrows point to TUNEL^+^ apoptosis cells (green). Nuclear was stained with DAPI (blue). Scar bar = 100 μm. **c** Quantification of apoptotic gut cells per section. Total apoptotic gut cells = TUNEL^+^ cells in intestinal bulb + TUNEL^+^ cells in mid-posterior intestine. n > 5 per group. **d** Western blot analysis of pro-Caspase3 and cleaved-aspase3 in two genotypes of zebrafish at 16 dpf. GAPDH is employed as a loading control. *p* indicates the significance (**p* < 0.05, ***p* < 0.01, ns, no significant difference).


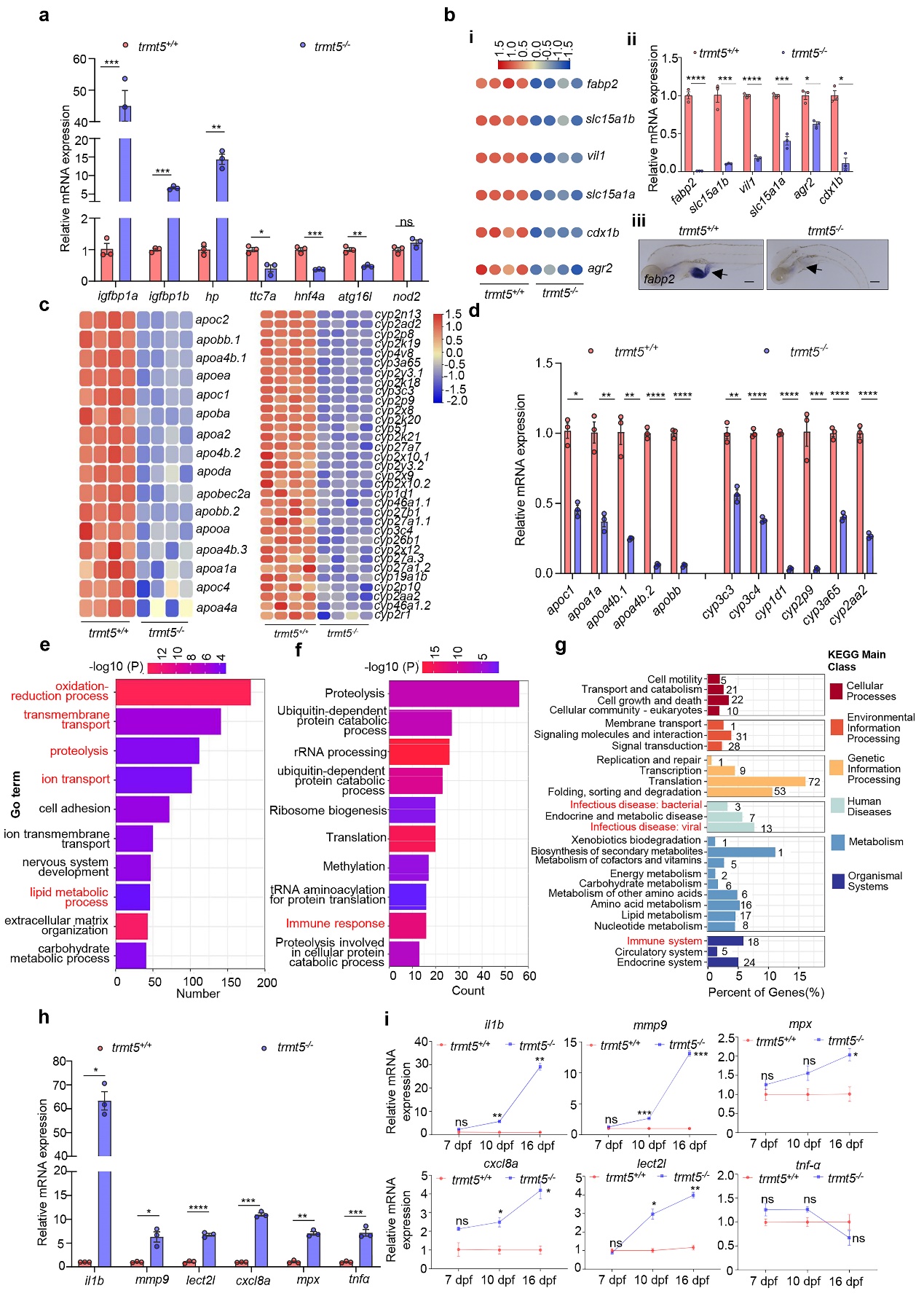


**Supplementary Figure 7. Dysregulated gene expression in *trmt5^-/-^*. a** The expression levels of representative IBD-related genes in the two zebrafish intestines at 16 dpf. **b** The expression of intestinal specific genes. i, the heatmap of intestinal specific genes by RNA-seq; ii, those genes’ relative expression in two intestines was verified by qRT-PCR. iii, Whole-mount in situ hybridization (WISH) analysis for *fabp2* at 16 dpf. Arrowhead indicates the gut. Scar bar = 200 μm. **c** Heatmaps of downregulated genes encoding apolipoproteins and enzymes of cytochrome P450 (CYP450) family related to intestinal metabolism. **d** qRT-PCR analysis of the representative genes was performed. **e** The top 10 GO terms of biological processes (BP) in the enrichment analysis of all down-regulated genes. **f** Top10 GO annotations of BP in the enrichment analysis of all upregulated genes. **g** KEGG enrichment bar plot of all upregulated genes. All enriched pathways were classified according to the KEGG database. KEGG main class represented primary classification, and KEGG_Subclass represented secondary classification. **h** Relative mRNA expression of pro-inflammatory genes was assessed using isolated gut RNAs at 16 dpf. **i** mRNA expression levels of representative pro-inflammatory genes were analyzed by qRT-PCR using whole-body total RNAs as templates at different time points. *p* indicates the significance (**p* < 0.05, ***p* < 0.01, ****p* < 0.001, *****p* < 0.0001, ns, no significant difference).


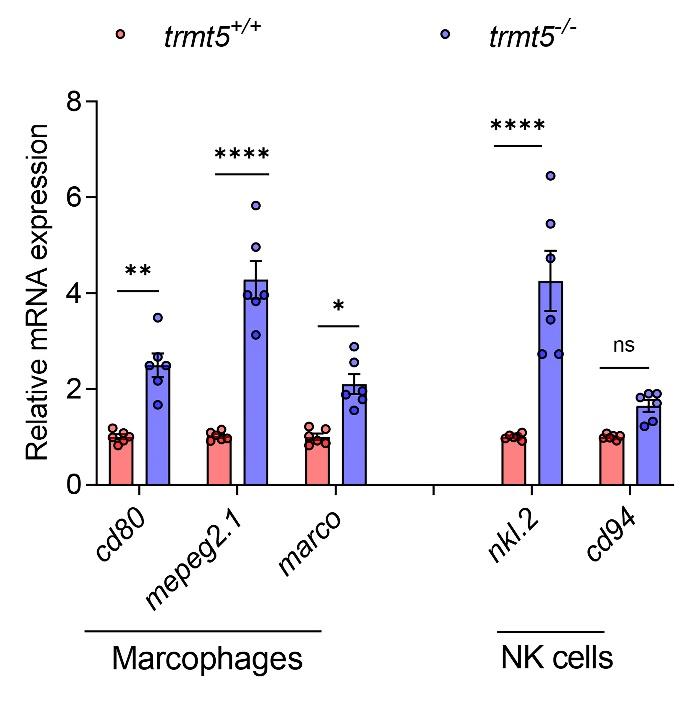


**Supplementary Figure 8. The mRNA expression of marker genes of some immune cells in intestine at 16dpf.**


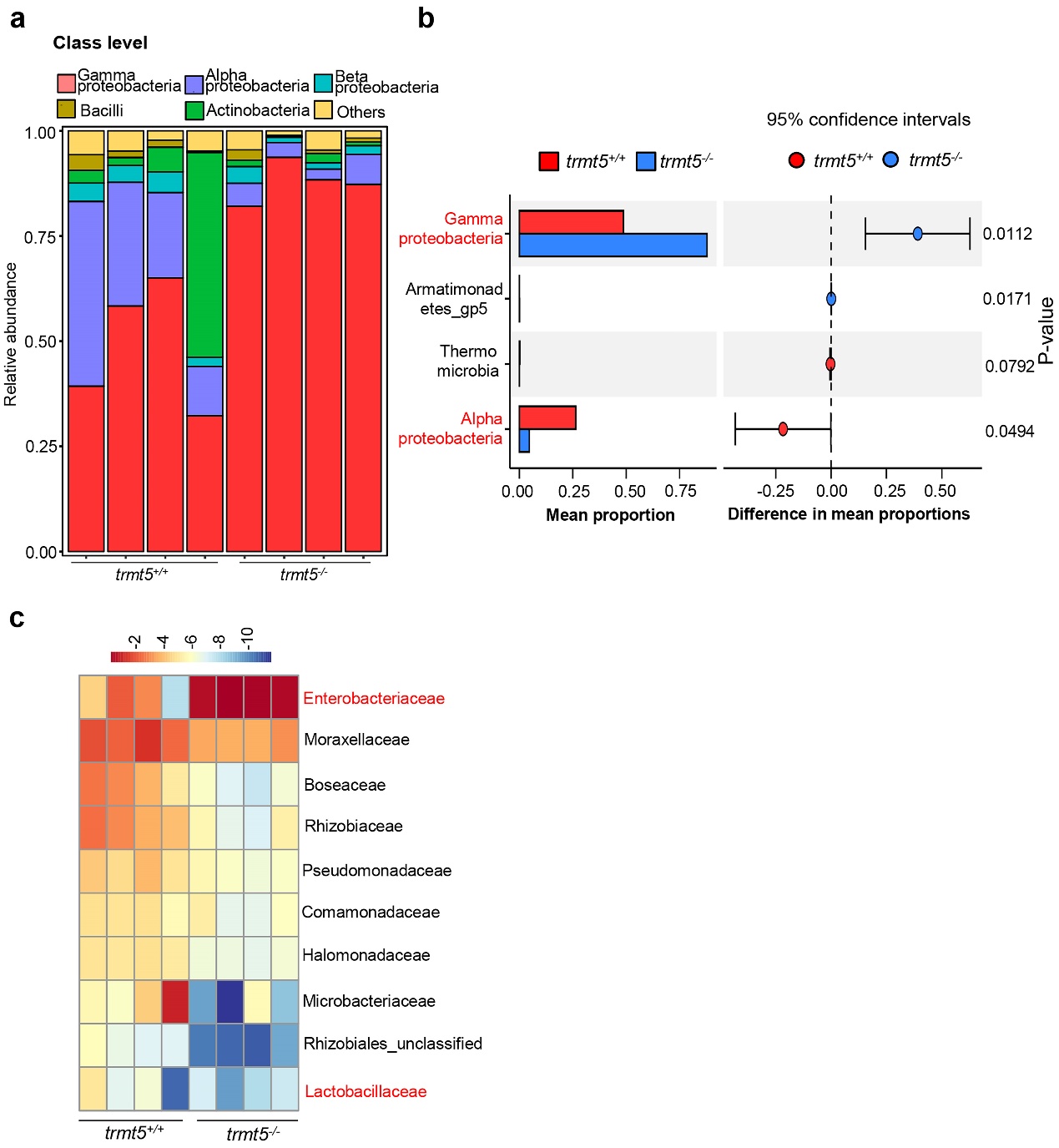


**Supplementary Figure 9. a** Metagenome analysis of the intestinal microbiota of *trmt5^-/-^* and *trmt5^+/+^* at the class levels. **b** STAMP analysis was performed to distinguish *trmt5^-/-^* from *trmt5^+/+^* at the class level. **c** Heatmap analysis of relative abundance of the top 10 most abundant bacteria families.


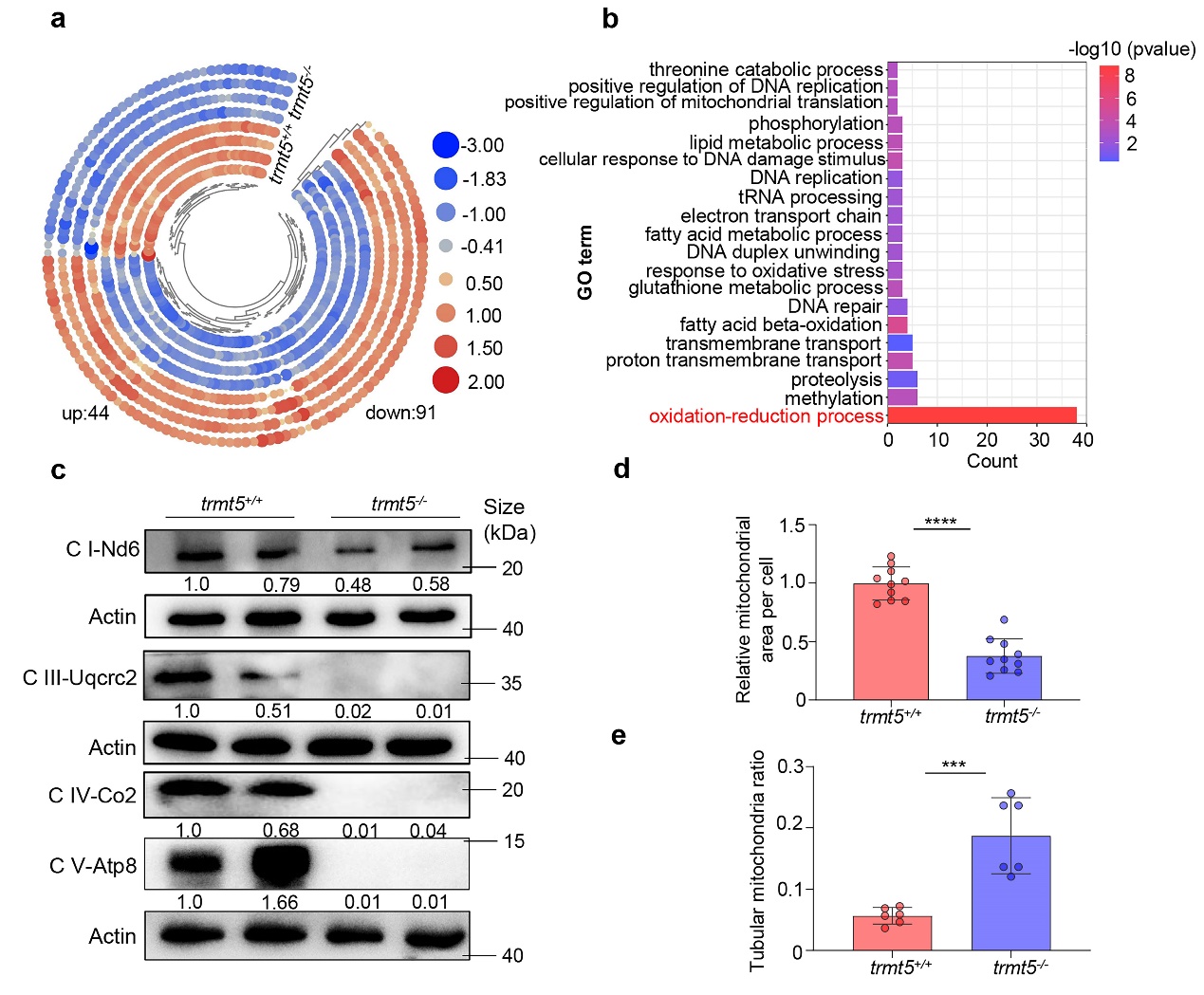


**Supplementary Figure 10. Mitochondrial dysfunction in *trmt5^-/-^* larvae. a** RNA-seq revealed a circular heatmap of mitochondrial-related DEGs. **b** The most significantly regulated GO terms of BP in the enrichment analysis of the mitochondrial-related DEGs. **c** Western bolting assays detected the expression level of oxidative phosphorylation (OXPHOS) subunits. Actin was adopted as a loading control. **d** Quantification of relative mitochondria area per cell in the intestine. **e** Quantification of the tubular mitochondria ratio.


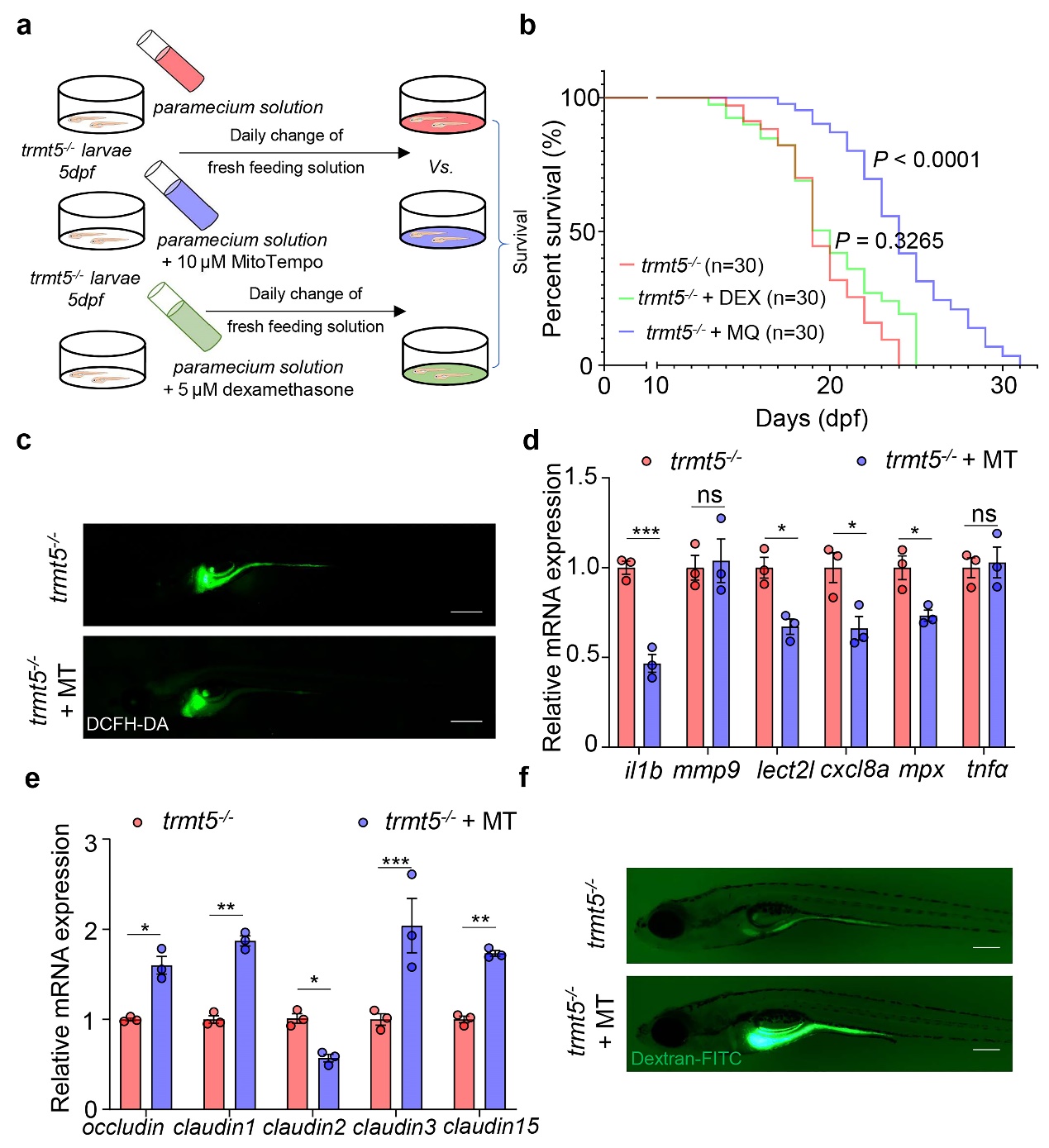


**Supplementary Figure 11. Drug treatments on *trmt5^-/-^* zebrafish. a** Schematic representation of the experimental design. The *trmt5^-/-^* larvae were fed with the same volume of paramecium solution with 10 μM MitoTempo (*trmt5^-/-^* + MT) or 5 μM dexamethasone (*trmt5^-/-^* + DEX) from 5 dpf. The solution was exchanged daily to maintain drugs concentrations, and the general condition and mortality of *trmt5^-/-^* larvae were monitored daily. MitoTempo, a novel cell-permeable antioxidant specifically targeted to mitochondria and subsequently eliminates mitochondrial superoxide. Dexamethasone, a widely used glucocorticoid derivative of cortisone with potent anti-inflammatory properties **b** The survival curves of *trmt5^-/-^* zebrafish treated with different drugs. **c** ROS production was decreased in *trmt5^-/-^* zebrafish by treating with MitoTempo at 16 dpf. **d** The mRNA expression of pro-inflammatory genes in two groups of zebrafish at 16 dpf. **e** The mRNA expression of tight junction-related genes in two groups of zebrafish at 16 dpf. **f** The swallow activity of *trmt5^-/-^* intestine was strengthened by MitoTempo treatment at 16 dpf.

**Supplementary Table 1. Sequences of primers for real-time PCR analysis**

| Gene | Forward primer | Reverse primer |
| --- | --- | --- |
| *trmt5* | TGTACGGGGCATGACAGAAC | TCCTGTATGTGGAGTCAATGGTG |
| *fabp2* | GCCCATGACAACCTGAAGAT | TGTCCTTGCGTGTGAAAGTC |
| *slc15a1b* | TTGGTTCCCCATGGCAAAGT | TTGGTTCCCCATGGCAAAGT |
| *vil1* | ACGCAGACTTCTGCATGTGA | AGCAGGAACACATCGCCTTT |
| *slc15a1a* | AGAACCGGCTGAGATGTAT | GAAGGCTGAAGGCTGGACT |
| *agr2* | GGTCACGATCCAAGAACAAG | CCATCAGGAGACAAGTGCTT |
| *apoc1* | TGCTGTACACACCAGAGG | TGGAAGGCGGTTTTGGTCTT |
| *apoa1a* | TATGCCCAGACCACCTCCC | GGCGGTTCAGAGCGGAGT |
| *apoa4b.1* | GAGTTCCAGAAAACTGTGAGTCCTCTAGCT | TCGTACAGAGAGATCAGCTGGTCTTTTAGG |
| *apoa4b.2* | TTGTGGTCTTTGCACTTGCT | TCATCTTGACGGTTTCCTCTG |
| *apobb* | GCT TGA AGG AAC CAG CAG TC | AGT TGG TGG TTG GCA TTA GC |
| *cyp3c3* | TCCAGACCTCTGGGAGTCTCCTAAT | GCATGAAGGCACACTGGTTGATCT |
| *cyp3c4* | TGGTCGCTGACCTGGAAGTGA | AAGGGGCCAGCCAGTCCTGT |
| *cyp1d1* | TCAACTTCGACACGAACTGTA | TGTGAACGATCTGGGAGTTG |
| *cyp2p9* | GTATCCTGGGATTCAAGCTAAAGTT | TATCCTCTGAAGTAGTCCTGGTCAC |
| *cyp3a65* | TCCATCGCAGAAGATGACGAC | GTTTTCCCCATGCTGTCAACC |
| *cyp2aa2* | GCCTTTTGTGGGAAACTTAC | AGCCAGTTGGATTGTATTGATGC |
| *il1b* | GGCTGTGTGTTTGGGAATCT | TGATAAACCAACCGGGACA |
| *cxcl8a* | TGTTTTCCTGGCATTTCTGACC | TTTACAGTGTGGGCTTGGAGGG |
| *lect2l* | TGTGTGTACTGATGGAGCCAC | CCGGACAAACGATCTGGCTT |
| *mmp9* | GCTCAACCACCGCAGACTAT | GTGCTTCATTGCTGTTCCCG |
| *mpx* | CTACATGGCACAAACGCTGAG | CTCGTCTTGAGTGAGCAGGTT |
| *tnfα* | CAGGGCAATCAACAAGATGG | TGGTCCTGGTCATCTCTCCA |
| *igfbp1a* | TTGAAGAGAGGTGACCCGTG | TTGGCTGTGGTTAGGCTCG |
| *igfbp1b* | CACCTGCTGAGCCTGAACAG | GAGAAGCTCAGTGTGACACGG |
| *atg16l* | AAACGTCACCGAGCAGACAG | CGAGCTGAACGAATTCACGG |
| Gene | Forward primer | Reverse primer |
| *nod2* | GCAAGGAGGGGGTTGATTGT | TCTGCATTCTTGCTGGCTCA |
| *hp* | CTGATGCTACAGCCTCTACGG | GATGTGTTCTGGAAGCCTGGA |
| *hnf4a* | TACTAGGAGCTGCCAAACGC | TTCTTACAGCCACACGGCTC |
| *ttc7a* | GAGACTGCTGTGTCTCGTCTG | ATGAACAACTCCCCTGCCTG |
